# Protocol of a randomized, double-blind, placebo-controlled study of the effect of probiotics on the gut microbiome of patients with gastro-oesophageal reflux disease treated with rabeprazole

**DOI:** 10.1186/s12876-022-02320-y

**Published:** 2022-05-20

**Authors:** Wenjun Liu, Yong Xie, Yingmeng Li, Longjin Zheng, Qiuping Xiao, Xu Zhou, Qiong Li, Ni Yang, Kexuan Zuo, Tielong Xu, Nong-Hua Lu, Heping Zhang

**Affiliations:** 1 Key Laboratory of Dairy Biotechnology and Engineering, Key Laboratory of Dairy Products Processing, Inner Mongolia Key Laboratory of Dairy Biotechnology and Engineering, Inner Mongolia Agricultural University, Ministry of Education, Ministry of Agriculture and Rural Affairs, Hohhot, 010018 China; 2 Department of Gastroenterology, The First Affiliated Hospital of Nanchang University, Nanchang, 330006 China; 3 State Key Laboratory of Innovative Medicines and High-Efficiency Energy-Saving Pharmaceutical Equipment, Nanchang, 330006 China; 4 Evidence Based Medicine Research Center, Jiangxi University of Chinese Medicine, Nanchang, 330004 China

**Keywords:** GERD, Probiotics, Proton pump inhibitors, Gut microbiome

## Abstract

**Background:**

For patients with gastro-oesophageal reflux symptoms, the preferred treatment is proton pump inhibitor (PPI) administration for approximately 8 weeks. However, long-term use of PPIs can cause gut microbiome (GM) disturbances. This study is designed to evaluate the effect of probiotics combined with a PPI on the GM and gastrointestinal symptoms of patients with gastro-oesophageal reflux disease (GERD).

**Method:**

This is a randomized, double-blind, placebo-controlled trial. A total of 120 eligible patients with GERD will be randomized into the experimental group or the control group. The treatment includes two phases: the initial treatment period lasts 8 weeks (weeks 1–8), and the maintenance treatment period lasts 4 weeks (weeks 9–12). During the initial treatment period, the experimental group will take rabeprazole and LiHuo probiotics, and the control group will take rabeprazole and a probiotic placebo; during the maintenance treatment period, the experimental group will take LiHuo probiotics, and the control group will take a probiotic placebo. The primary measure is the change in the GM. The secondary measures are the Reflux Disease Questionnaire (RDQ) score, Gastrointestinal Symptom Rating Scale (GSRS) score, faecal metabolome (FM), body mass index, Los Angeles grade of oesophagitis, adverse event (AE) rate and treatment compliance. Each outcome indicator will be assessed at day 0 (before administration), day 28 and/or 56 (during administration), and day 84 (end of administration) to reveal intragroup differences. AEs will be monitored to assess the safety of LiHuo probiotics.

**Discussion:**

This will be the first trial to use the intestinal flora metagene method to analyse the effects of probiotics on patients with GERD receiving long-term PPI treatment. The goal is to provide evidence for the use of probiotics to reduce intestinal flora disorders and other symptoms of gastrointestinal discomfort in patients with GERD who have used PPIs for a long period.

***Trial registration*:**

Chinese Clinical Trial Registry (ChiCTR) (NO. ChiCTR2000038409). Registered on November 22, 2020, http://www.chictr.org.cn/showproj.aspx?proj=56358.

**Supplementary Information:**

The online version contains supplementary material available at 10.1186/s12876-022-02320-y.

## Background

With the widespread availability of proton pump inhibitors (PPIs) in digestive diseases, PPIs are used in the treatment of GERD and gastric or duodenal ulcers and as part of *Helicobacter pylori* eradication. PPIs are the most widely prescribed class of drugs in the United States. In 2012, 14.9 million United States patients received 157 million prescriptions [1]. From 2006 to 2010, PPIs accounted for more than $10 billion in estimated annual healthcare costs in the United States [2]. Based on the French National Health Data System (SNDS), there were 15,388,419 people who used PPIs at least once in 2015, accounting for 29.8% of the French adult population. The average treatment time was 40.9 days, but 4.1% of users received continuous PPI treatment for more than 6 months [3]. GERD refers to the reflux of gastroduodenal contents into the oesophagus that causes symptoms such as acid reflux and heartburn. Global population-based research results show that the prevalence of GERD ranges from 2.5% in China to 51.2% in Greece [4]. The prevalence of GERD symptoms occurring at least once a week is 13% and is higher in Western countries [5]. The prevalence of GERD symptoms in the Asia–Pacific region is increasing [6]. In a survey of 71,812 American participants, 32,878 (44.1%) people had experienced GERD symptoms in the past, and 23,039 (30.9%) people had experienced GERD symptoms in the past week; 35.1% of people who had symptoms of GERD were currently receiving medication (55.2% of them used PPIs). Among the 3229 participants who took PPIs, 54.1% had persistent GERD symptoms [7].

Moderate alterations to the upper and distal gut microbiota were observed in patients who used PPIs [8], and changes in the microbiota of the gastrointestinal tract are also associated with PPIs. Previous studies have shown that PPI use results in an increase in multiple taxa from the orders *Lactobacillales* (e.g., *Enterococcaceae* and *Streptococcaceae*) and *Bacillales* (e.g., *Staphylococcaceae*), as well as from the families *Pasteurellaceae* and *Enterobacteriaceae,* and a decrease in the families *Bifidobacteriaceae*, *Ruminococcaceae*, and *Lachnospiraceae* and the class *Mollicutes*. PPIs have the most significant impact on the microbiota in addition to antimicrobials according to the literature [9]. A study indicated that the GM of rheumatoid arthritis (RA) patients who were receiving PPIs was different, as *Streptococcus* was enriched in RA patients who received PPIs. The gut microbiota of PPI users could be modified by the production of virulence factors. This featured microbial function was positively correlated with the relative abundance of *Streptococcus* [10]. Frequent use of PPIs was associated with an increased risk of RA in women, with a higher risk observed in people with a longer PPI treatment period [11]. Researchers of one team observed positive effects of a multispecies probiotic in patients undergoing long-term PPI therapy. The richness of *Stomatobaculum* in the microbiome was decreased and that of *Bacillu*s increased during the intervention, and gastrointestinal quality of life simultaneously showed significant improvements. However, only sparse evidence can support the routine use of probiotics during PPI therapy [12].

In a clinical study on children, the incidence of gut microbial dysbiosis in children treated with PPIs combined with probiotics was only 6.2%, and 56.2% of children who used PPIs and placebo after 12 weeks developed gut microbial dysbiosis. Taking probiotics reduces the incidence of gut microbial dysbiosis in children receiving PPI treatment [13]. Additionally, probiotics can significantly reduce the frequency of reflux, promote gastric emptying, and improve reflux symptoms in infants [14, 15].

In a systematic review on the association between GERD and probiotics, five of eleven studies indicated that probiotics are beneficial for reflux symptoms; three studies indicated that probiotics can reduce reflux symptoms; one study reported probiotic-induced improvements in reflux or heartburn; dyspepsia symptoms improved in five studies; and other upper gastrointestinal symptoms were improved in nine studies, with such symptoms including nausea (three studies), abdominal pain (five studies), and gas-related disorders (four studies) such as belching, gurgling, and burping. Probiotics can effectively reduce the frequency and severity of reflux, heartburn and dyspepsia in adults suffering from GERD [16]. In the observation of reflux oesophagitis, compared with patients using placebo and esomeprazole, patients who use probiotics combined with esomeprazole for 8 weeks have significantly lower Gastrointestinal Symptom Rating Scale (GSRS) diarrheic comprehensive scores and a significantly higher negative rate of small intestinal bacterial overgrowth [17].

However, there are still few clinical studies on adult gastro-oesophageal reflux patients who use PPIs and probiotics to improve their symptoms compared with those who use a PPI only, and there is no study comparing PPI treatment alone and PPI treatment combined with probiotics in terms of the analysis of intestinal microbiota by metagenomics. Therefore, this study is designed to observe the change in reflux symptoms, the gastrointestinal symptoms of patients with long-term use of PPIs, the metagenomic change in intestinal microbial, and the change after probiotic intervention.

## Methods and design

### Design

**Fig. 1:**
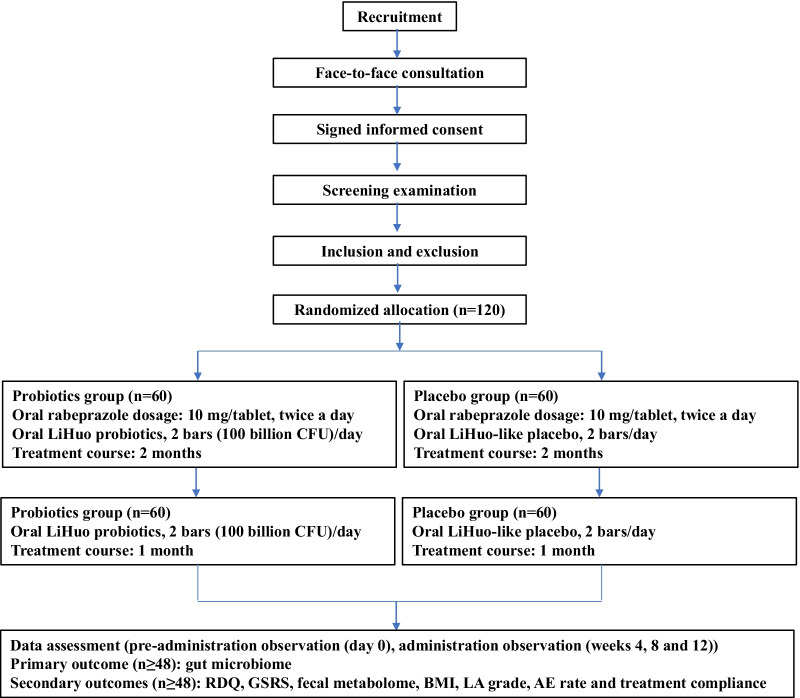
Flow chart of the protocol

This is a randomized, double-blind, placebo-controlled clinical trial. The protocol was prospectively registered in the Chinese Clinical Trial Registry (ChiCTR) (No. ChiCTR 2,000,038,409) (Additional file 1: Appendix 1) and approved by the Ethics Committee of the First Affiliated Hospital of Nanchang University (Approval No. IIT [2020] EC 003-2) (Additional file 1: Appendix 2). This protocol was developed based on the Standard Protocol Items: Recommendations for Interventional Trials (SPIRIT). The study process is shown in Fig. 1.

### Volunteer recruitment

Posters will be disseminated at the hospital to recruit participants. During their appointment at the Department of Gastroenterology in the First Affiliated Hospital of Nanchang University, volunteers who are interested in the study will undergo preliminary evaluations based on the inclusion and exclusion criteria. Additionally, the investigator will explain the purpose, process, and potential benefits and risks of this study, and the volunteers may choose to participate by signing the informed consent form. Next, the volunteers will undergo further screening to determine their eligibility. Eligible participants will receive the study intervention and follow-up. The following steps describe the formal intervention and follow-ups (Table 1).

**Table 1:** Study and follow-up schedule

Item^1,10^		Screening	Baseline	Initial treatment		Maintenance treatment
		Visit 0	Visit 1	Visit 2	Visit 3	Visit 4
		Days −3 to 0	Day 0	Week 4	Week 8	Week 12
Basic demographics		X				
Vital signs, physical examination		X				
Allergies, medical history^2^, medication history^3^, surgical history^4^		X				
Smoking and drinking		X				
Gastroscopy		X^5^				X^6^
Abdominal ultrasound B^7^		X				
Serum human chorionic gonadotropin (hCG)		X				
ECG		X				X
Blood test, urine analysis, stool test (including occult blood)		X				X
Liver/kidney function^8^		X				X
Signed informed consent		X				
Inclusion/exclusion criteria verification			X			
Primary outcome measure	GM^11^ (metagenomics)	X		X	X	X
Secondary outcome measures	RDQ	X	X	X	X	X
	GSRS		X	X	X	X
	Faecal metabolomics^11^	X		X	X	X
	BMI ^9^		X	X	X	X
	LA grade	X				X
Safety measures	AEs			X	X	X
	SAEs			X	X	X
Compliance verification			X	X	X	
Concomitant medications		X	X	X	X	

^1^Visit window: 0 days for visit 1; ± 2 days for visits 2, 3, and 4

^2^Medical history: current and past illnesses, including peptic ulcer, gastritis, oesophagitis, cardiovascular and cerebrovascular diseases, cancer, and mental illness

^3^Medication history: PPI, histamine H2 antagonists, antibiotics, probiotics, prokinetics, gastric mucosal protectors, and other drugs for gastro-oesophageal reflux (including herbs)

^4^Surgical history: gastrointestinal surgeries, including gastro-oesophageal and duodenal surgeries

^5^Performance of gastroscopy in domestic tertiary or higher hospitals in the past 3 months

^6^Gastroscopy at visit 4, LA grades at visit 0 and visit 4 referring to patients with oesophagitis (LA-A, LA-B or LA-C)

^7^Abdominal ultrasound B: to examine the abdominal organs to screen for upper abdominal malignancies

^8^Liver and kidney tests: ALT, AST, total bilirubin, blood urea nitrogen, Scr

Weight and height will be measured in the morning

^10^For the abdominal B-ultrasound and ECG evaluations performed at visit 0, examination results from the past 3 months are acceptable; examination results from the past 1 week are acceptable for blood HCG, blood, routine urine and stool (including occult blood) tests, and liver/kidney function tests

^11^The detection of GM will be conducted within 2 months after the end of the follow-ups, and the detection of FM will be performed within 2 months of when data analysis determines that the probiotics have a significant effect on GM

### Inclusion criteria

1. Consenting (Additional file 1: Appendix 3) patients with GERD or one of its signs after gastroscopy:
  1. In the past 3 months, gastroscopy performed at domestic tertiary hospitals has shown oesophagitis (LA-A, LA-B or LA-C);
  2. In the past 3 months, gastroscopy performed at domestic tertiary hospitals has not revealed oesophagitis, but there are symptoms such as heartburn, acid reflux, and poststernal burning pain; in addition, the RDQ score is ≥ 12 [18];
2. The subject’s age is 18–65 years (inclusive), male or female.

### Exclusion criteria

1. Use of GERD-related drugs such as acid inhibitors, antacids, prokinetics, gastric mucosal protectors, and herbs (see Additional file 1: Appendix 4) or probiotics and probiotic-related preparations in the last 2 weeks;
2. Any of the following conditions:
  - Liver insufficiency, defined as alanine aminotransferase (ALT) or aspartate aminotransferase (AST) > 2 × the upper limit of normal (ULN);
  - Renal insufficiency, defined as serum creatinine (Scr) > 1 × ULN;
  - Heart failure or electrocardiogram (ECG) abnormalities;
3. Peptic ulcer and bleeding, oesophageal gastric varices, or upper gastrointestinal malignancies confirmed by endoscopy at tertiary hospitals in China in the last 3 months;
4. Myocardial infarction, stroke, or malignant tumour;
5. History of gastro-oesophageal or duodenal surgery;
6. Plans to become pregnant or father a child in the near future, or pregnancy or breastfeeding in women.
7. Inability to cooperate, such as an inability to understand the informed consent form or unwillingness to provide personal information;
8. Allergies to the study drug (rabeprazole) or probiotics;
9. Oesophagitis caused by gastric retention and pyloric obstruction.

### Randomization and blinding

According to their order of participation in the study, volunteers who sign the informed consent form will be assigned a screening number (e.g., 010,001, 010,002, 010,003, 010,004, 010,005); then, they will receive a serially unique number (e.g., G1001, G1002, G1003, G1004, G1005) according to their screening result. For each of these unique numbers, a random sequence will be generated by the computer software R 4.1.0 and used to randomly assign the unique number (representing a volunteer) to the probiotic group or placebo group. During the study, the subjects and study staff responsible for sample distribution, data collection, data collation and analysis will be blinded to the random sequence, which will be maintained by two independent project managers and unblinded only in the case of important safety issues, and the final data analysis will be completed. Moreover, an independent project administrator will label the probiotics or placebo packs with unique numbers corresponding to random sequences in advance to achieve allocation concealment. According to the order of randomization, the distributor will distribute the treatment packs to the corresponding volunteers.

### Study intervention

The study treatment consists of two phases: an initial treatment period lasting 8 weeks (weeks 1–8) and a maintenance treatment period lasting 4 weeks (weeks 9–12). The patients will be randomized into two groups: a control group and an experimental group. The participants will not know the group to which they have been assigned.

### Intervention strength and management

Rabeprazole: 10 mg/tablet, from Eisai China, Inc (Tokyo, Japan).

LiHuo Probiotics powder: LiHuo probiotics powder containing strains such as *Lactobacillus casei Zhang*, *Bifidobacterium lactis* V9, and *Lactobacillus plantarum* P9, 2 g/bar, each bar containing active probiotics (≥ 50 billion CFU), were obtained from Jiangzhong Pharmaceutical Company Limited (China). During the manufacturing of LiHuo probiotics powder, *Lactobacillus casei Zhang*, *Bifidobacterium lactis* V9, and *Lactobacillus plantarum* P9 were mixed according to concentrations of ≥ 5 billion CFU/g, ≥ 15 billion CFU/g, and ≥ 5 billion CFU/g, respectively.

Probiotic placebo powder: The ingredients are maltodextrin, orange powder, and maltitol, and there are no active ingredients. The appearance, packaging, storage method, and dosing are the same as those of the LiHuo probiotics from Jiangzhong Pharmaceutical Company Limited (China).

The rabeprazole, LiHuo probiotics, and probiotic placebo will be stored in a cool, dry place away from light. Refrigerated storage will retain the maximum activity of the product. Each patient will receive a sample that corresponds to his or her unique number. All remaining study drugs and empty packages will be collected at the end of the study.

The study will have three phases: the screening period, the initial treatment period, and the maintenance treatment period. The interventions in each phase are described below:

1. Screening: No intervention.
2. Initial treatment (weeks 1–8):
  Control group: rabeprazole and probiotic placebo. The samples will be distributed to the patients every month.
  Experimental group: rabeprazole and probiotics. The samples will be distributed to the patients every month.
  Rabeprazole dosage: 10 mg/tablet twice a day before meals;
  Dosage of probiotic placebo or LiHuo probiotics: 2 bars, once a day, with or without water (< 40 °C), after meals. Thus, the daily dosage of probiotics is more than 100 billion CUF. Participants who take antibiotics should wait 2 h before taking the probiotic placebo or LiHuo probiotics.
3. Maintenance treatment (weeks 9–12):

Control group: probiotic placebo. The samples will be distributed to the patients every month.

Experimental group: probiotics. The samples will be distributed to the patients every month.

Dosage of probiotic placebo or LiHuo probiotics: 2 bars, once a day, with or without water (< 40 °C), after meals. Participants who take antibiotics should wait 2 h before taking the probiotic placebo or LiHuo probiotics.

### To minimize confounding interventions

1. Prohibited treatments during the study.
  1. Probiotics, prebiotics, and foods containing probiotics (such as yogurt) other than those used in this study;
  2. Drugs for gastro-oesophageal reflux, such as acid inhibitors, antacids, gastric mucosal protectors, prokinetics, and equivalent drugs [19, 20] (see Additional file 1: Appendix 4) other than the study drug;
2. Antibiotics and probiotics should be taken 2 h apart;
3. Participants will be instructed to avoid spicy foods and to abstain from smoking or drinking during the study.

### Primary outcome

Based on faeces, the GM from day 0 (week 0), day 28 (week 4), day 56 (week 8), and day 84 (week 12) will be evaluated. For the GM assessment, DNA will be extracted from the stool samples with the QIAamp Fast DNA Stool Mini Kit (Qiagen, Hilden, Germany) following the manufacturer’s instructions, and the DNA quality will be examined by agarose gel electrophoresis and a Nanodrop spectrophotometer. Shotgun metagenomic sequencing will be performed on all samples by using an Illumina HiSeq 2500 instrument. Libraries will be constructed from DNA fragments ~ 300 bp in length and paired-end reads will be generated by sequencing 150-bp lengths in the forward and reverse directions. Meanwhile, the metagenomic analysis will include several components: the analysis of alpha and beta diversities in each group to understand whether the differences in the microbiota compositions of groups are significant, as well as the taxonomic characteristics of the study and control groups at the phylum, genus, and species levels to identify specific genes related to the individual differences in GERD. Metagenomic biological pathway analysis will be used to evaluate the effect of probiotics on the function of gut metagenomics in patients with GERD and, hopefully, to explore the metagenomic biological pathways contributing to the mechanism of probiotics in the treatment of GERD.

### Secondary outcome

1. RDQ score: The RDQ (see Additional file 1: Appendix 5) includes 4 symptoms: heartburn, acid reflux, regurgitation, and noncardiac chest pain. Each symptom is rated on a 0–5 scale based on its frequency and severity in the last week. The total score is 40, and a higher score is associated with more severe symptoms. RDQ ≥ 12 indicates GERD [18, 21]. The RDQ will be used to evaluate reflux-related symptom scores on day 0 (week 0), day 28 (week 4), day 56 (week 8), and day 84 (week 12).
2. GSRS score: The GSRS (see Additional file 1: Appendix 6) is a self-rating scale that includes 15 items in 5 subscales (abdominal pain, acid reflux, dyspepsia, constipation, and diarrhoea). Each item is rated on a Likert scale ranging from 0–3 (none, mild, moderate, mild) based on the severity of symptoms in the last week. The total score is 45, and a higher score is associated with more severe symptoms [22–24]. The GSRS will be used to evaluate gastrointestinal disease symptom scores on day 0 (week 0), day 28 (week 4), day 56 (week 8), and day 84 (week 12).
3. Faecal metabolome (FM): Based on the faeces, FM from day 0 (week 0), day 28 (week 4), day 56 (week 8), and day 84 (week 12) will be evaluated. Stool samples will be extracted using the protein precipitation method, and the supernatant will be transferred to sample vials for mass spectrometry combined with liquid chromatography (LC–MS/MS) analysis. The original data will be subjected to peak alignment, retention time correction, and peak area extraction through the XCMS-Plus program. The structure of metabolites will be identified by accurate mass matching (< 5 ppm) and two-level spectrum matching, and the METLIN database will be retrieved. Missing values > 50% in the group will be deleted, and the data will be normalized. Multidimensional statistical analysis will then be carried out, including unsupervised principal component analysis (PCA), supervised partial least squares discriminant analysis (PLS-DA) and potential differential metabolite analysis. Metabonomic analysis could further identify the potential differential metabolites of probiotics in the treatment of GERD, and correlation analysis between the gut microbiota and metabolites could be performed.
4. Change in the Los Angeles (LA) grade of oesophagitis: According to gastroscopy, the change in the LA grade of oesophagitis will be evaluated.
5. Body mass index (BMI): BMI = weight (kg)/height (m)^2^. BMI will be measured in the morning using the same weight and height scales at each visit. Participants will be instructed to wear light clothing and take off their shoes (standing barefoot or wearing socks no thicker than 0.1 cm) during the weight and height measurements.
6. AE rate: all the AEs collected as described below will be used to calculate the AE rate in each group.
7. Treatment compliance will be monitored as described in the “Volunteer compliance monitoring” section. Although efforts have been made to promote treatment compliance, there may still be differences between the two groups due to AEs.

### Safety evaluation

1. AEs will be evaluated with blood tests, urine analysis, and stool tests (> 2 × ULN or < 2 × LLN); liver tests (ALT, AST, total bilirubin); renal tests (Scr, blood urea nitrogen); and ECG. AEs may be related or unrelated to the study interventions.
  At each visit, the patients will be asked about any AEs with nondirective questions. Patients may also voluntarily report AEs.
2. Severe adverse events (SAEs) are events that occur after the study interventions are provided and result in hospitalization, prolonged hospitalization, disability, impaired ability to work, threats to the patient’s life, or death. All SAEs must be reported to the Ethics Committee of the hospital and the sponsor. Participants with SAEs will discontinue the interventions.
3. Treatment-related AEs must meet the following criteria: a reasonable temporal/causal relationship with the treatment; side effects unrelated to comorbidities or concomitant medications; improvement or resolution of AE symptoms after treatment discontinuation; and causality can be explained with a pharmacological, biological, or phenomenological mechanism.

Drug-related AEs: possible adverse reactions experienced while taking rabeprazole include allergies (rash, itching, urticaria), abnormal blood test results, abnormal biochemical parameters, high blood pressure, gastrointestinal symptoms (constipation, diarrhoea, abdominal distension, nausea, lower abdominal pain, bitter taste in the mouth), headache, dizziness, palpitations, fever, fatigue, hypoesthesia, dry mouth, blurred vision, and vomiting. Possible serious adverse reactions include shock, interstitial nephritis, liver dysfunction, jaundice, anaemia, and hyponatremia. Possible adverse reactions to probiotic ingestion include diarrhoea, constipation, and weight loss.

### Volunteer compliance monitoring

For the initial treatment (weeks 1–8), the sample package will include rabeprazole and probiotics or rabeprazole and the probiotic placebo; for the maintenance treatment (weeks 9–12), the sample package will include probiotics or the probiotic placebo. After the start of the study, the volunteers will receive a sample package at each monthly visit (package 1 at visit 1, package 2 at visit 2, and package 3 at visit 3) and will be required to return the package at the next visit such that the remaining rabeprazole, probiotics, or probiotic placebo doses can be counted to calculate the compliance level based on the equation below. Compliance ≥ 80% indicates good compliance.

Compliance = [actual amount taken (packets)]/[(amount prescribed (packets)] × 100%

To improve compliance, the study staff will use push messages via a WeChat group established for this study to remind the volunteers to take the sample as scheduled. All volunteers who complete the follow-up will receive 100 yuan as compensation. Moreover, rabeprazole will be provided at no cost during the study.

### Sample size

This is a superiority study. Based on pilot case observations and expert feedback, the major indicator Chao1 index (α-diversity) at week 12 is expected to differ by 145 between the two groups, with a standard deviation of 230 [25]. Assuming *α* = 0.025 and *β* = 0.20, we calculated that the sample size should be at least 47.9 in each group [26]. If the lost-to-follow-up rate is no more than 20%, the sample size should be at least 57.5 in each group. Moreover, Shannon’s index (α-diversity) of the primary measure at week 12 is expected to differ by 0.9 between the two groups, with a standard deviation of 0.8. Thus, the sample size should be at least 15.0 in each group. If the lost-to-follow-up rate is no greater than 20%, the sample size should be at least 18.0 in each group. The larger value (57.5) is used as the calculated sample size. Based on the above assumptions and our budget, we will recruit a total of 120 participants for this study, with 60 participants in each group.

### Data collection and collation

All study participants will be assigned a study ID. Electronic data will be stored in a secure password-protected database. All paper data collected will be stored at the site, and only the study team will have access to the data. The study results will be independently analysed at the end of the study. No interim analysis is planned. The GM and metabolomics data detected from stool samples will be analysed by the Key Laboratory of Dairy Biotechnology and Engineering, Ministry of Education. All records that contain names or other personal identifiers referring to a specific volunteer, such as ID number and informed consent forms, will be stored separately from the study records. The database will be password protected by the data management team (DMT).

### Statistical analysis

First, the intention-to-treat (ITT) analysis will be conducted between the two groups, including all the intended participants who do or do not complete the follow-up. Then, per-protocol (PP) analysis will be conducted in the four-subgroup datasets according to compliance (100%, ≥ 80%, 60–80%, and < 60%) to validate the robustness of the results. The datasets will be analysed as described below.

With a randomized block design, the nonparametric rank sum test and Friedman M test will be performed to analyse the difference in each outcome measure at each time point (see “Intervention Outcome Measures” for details) between the two groups, including the GM analysis, RDQ score, GSRS score, faecal metabolomics, and BMI. The significance level will be set to *P* = 0.05. Next, the paired Wilcoxon signed-rank test will be performed for pairwise intragroup comparisons to analyse the change trends for each outcome measure before, during, and after the probiotic intervention. The significance level will be set at *P* = 0.05/[k(k-1)/2], where k is the number of groups in the comparison. For analysis of the oesophagitis grade, the diagnosis of LA-B at baseline and LA-A at week 12 indicates that the patient's oesophagitis grade will have decreased by one level. If the grade is LA-C at baseline and LA-A at 12 weeks, the patient’s oesophagitis grade will have decreased by 2 levels. The number of oesophagitis grades reduced by 1 and 2 levels in each group will be calculated. Then, a chi-square (*χ*^2^) test will be performed. AE rates in the two groups will be compared by the *χ*^2^ test or Fisher’s exact probability test based on the occurrence frequency of AE, and the difference in treatment compliance will be also examined by the *χ*^2^ test.

## Discussion

Long-term use of PPIs in the treatment of patients with GERD affects their quality of life and causes discomfort and new problems such as dysbiosis of the gut microbiota. Probiotics may provide solutions and answers to patients’ questions regarding these issues. The amount of research in this area is limited, which is why this study was designed.

The probiotics used in the research were composed of *Lactobacillus casei Zhang*, *Bifidobacterium lactis* V9, and *Lactobacillus plantarum* P9. *Lactobacillus casei Zhang* can colonize the intestine, improve the structure of intestinal flora, affect the abundance of intestinal microbes, increase the metabolism of short-chain fatty acids, inhibit the formation of secondary bile acids, and have a positive and lasting impact on the intestinal microbiota of subjects [27]. Compared with *Lactobacillus acidophilus*, *Lactobacillus casei Zhang* has a stronger ability to improve the intestinal mucosal barrier and promote the expansion of beneficial metabolites, especially short-chain fatty acids and niacinamide [28]. *Bifidobacterium lactis* V9 has a stable colonization ability in the patient’s intestine. After 4 weeks of intervention, the structure and metabolism of the patient’s intestinal flora will change significantly, which can effectively regulate the patient’s gut microbial dysbiosis [29]. *Lactobacillus plantarum* P9 belongs to the genus *Lactobacillus*, which is one of the most widely studied probiotics. Many reports have demonstrated the improvement in gastrointestinal symptoms and the intestinal microbiota with *Lactobacillus* treatment.

Based on the effects of these three bacteria on the regulation of intestinal flora in the past, we suspect that the LiHuo probiotics used in this study can play a good regulatory role and provide a more comfortable and safe medication experience in patients who have used PPIs for a long time.

In addition, if PPI has detrimental effects on gastrointestinal tract homeostasis, concurrent probiotic administration with PPI may be more suitable rather than post PPI use. It may be interesting to confirm the effects of concurrent probiotic administration with PPI use on the GERD and even study the pros and cons between the two administrations of probiotics using along with PPI and post-PPI.

## Supplementary Information

**Additional file 1: Appendix 1.** Trial registration dataset.

**Additional file 2: Appendix 2.** Copy of Ethical Approval Document with translated English version.

**Additional file 3: Appendix 3.** Patient informed consent form.

**Additional file 4: Appendix 4.** Common Drugs Taken for Gastroesophageal Reflux SymptomsAppendix.

**Additional file 5: Appendix 5.** Reflux Disease Questionnaire.

**Additional file 6: Appendix 6.** Gastrointestinal Symptom Rating Scale.

**Additional file 7: Appendix 7.** National Natural Science Foundation of China with translated English version

## Data Availability

Data sharing is not applicable to this trial, as no database was generated or analysed for the current study. When the study is completed, its results will be released to the public, volunteers, and the general medical community through the publication of journal articles, with all the related data made available.

## Abbreviations

AEs: Adverse events
ALT: Alanine aminotransferase
AST: Aspartate aminotransferase
BMI: Body mass index
ECG: Electrocardiogram
FM: Faecal metabolome
GM: Gut microbiome
GSRS: Gastrointestinal Symptom Rating Scale
GERD: Gastroesophageal reflux disease
PPIs: Proton pump inhibitors
RDQ: Reflux disease questionnaire
ULN: Upper limit of normal
Scr: Serum creatinine
SAEs: Severe adverse events

## References

[1] Elias E, Targownik LE (2019). The clinician's guide to proton pump inhibitor related adverse events. Drugs.

[2] Boster J, Lowry LE, Bezzant ML, Kuiper B, Surry L (2020). Reducing the inappropriate use of proton pump inhibitors in an internal medicine residency clinic. Cureus.

[3] Lassalle M, Le Tri T, Bardou M, Biour M, Kirchgesner J, Rouby F (2020). Use of proton pump inhibitors in adults in France: a nationwide drug utilization study. Eur J Clin Pharmacol.

[4] Katzka DA, Kahrilas PJ (2020). Advances in the diagnosis and management of gastroesophageal reflux disease. BMJ.

[5] Eusebi LH, Ratnakumaran R, Yuan Y, Solaymani-Dodaran M, Bazzoli F, Ford AC (2018). Global prevalence of, and risk factors for, gastro-oesophageal reflux symptoms: a meta-analysis. Gut.

[6] Fock KM, Talley N, Goh KL, Sugano K, Katelaris P, Holtmann G (2016). Asia-Pacific consensus on the management of gastro-oesophageal reflux disease: an update focusing on refractory reflux disease and Barrett's oesophagus. Gut.

[7] Delshad SD, Almario CV, Chey WD, Spiegel BMR (2020). Prevalence of gastroesophageal reflux disease and proton pump inhibitor-refractory symptoms. Gastroenterology.

[8] Macke L, Schulz C, Koletzko L, Malfertheiner P (2020). Systematic review: the effects of proton pump inhibitors on the microbiome of the digestive tract-evidence from next-generation sequencing studies. Aliment Pharmacol Ther.

[9] Perry IE, Sonu I, Scarpignato C, Akiyama J, Hongo M, Vega KJ (2020). Potential proton pump inhibitor-related adverse effects. Ann N Y Acad Sci.

[10] Kim JW, Jeong Y, Park SJ, Jin H, Lee J, Ju JH (2021). Influence of proton pump inhibitor or rebamipide use on gut microbiota of rheumatoid arthritis patients. Rheumatology (Oxford).

[11] Yuan J, Zhang C, Sparks JA, Malspeis S, Tsoi KK, Kim JH (2020). Regular use of proton pump inhibitor and risk of rheumatoid arthritis in women: a prospective cohort study. Aliment Pharmacol Ther.

[12] Horvath A, Leber B, Feldbacher N, Steinwender M, Komarova I, Rainer F (2020). The effects of a multispecies synbiotic on microbiome-related side effects of long-term proton pump inhibitor use: a pilot study. Sci Rep.

[13] Belei O, Olariu L, Dobrescu A, Marcovici T, Marginean O (2018). Is it useful to administer probiotics together with proton pump inhibitors in children with gastroesophageal reflux?. J Neurogastroenterol Motil.

[14] Indrio F, Di Mauro A, Riezzo G, Civardi E, Intini C, Corvaglia L (2014). Prophylactic use of a probiotic in the prevention of colic, regurgitation, and functional constipation: a randomized clinical trial. JAMA Pediatr.

[15] Indrio F, Riezzo G, Raimondi F, Bisceglia M, Filannino A, Cavallo L (2011). *Lactobacillus reuteri* accelerates gastric emptying and improves regurgitation in infants. Eur J Clin Invest.

[16] Cheng J, Ouwehand AC (2020). Gastroesophageal reflux disease and probiotics: a systematic review. Nutrients.

[17] Sun QH, Wang HY, Sun SD, Zhang X, Zhang H (2019). Beneficial effect of probiotics supplements in reflux esophagitis treated with esomeprazole: a randomized controlled trial. World J Gastroenterol.

[18] Shaw M, Dent J, Beebe T, Junghard O, Wiklund I, Lind T (2008). The reflux disease questionnaire: a measure for assessment of treatment response in clinical trials. Health Qual Life Outcomes.

[19] MacFarlane B (2018). Management of gastroesophageal reflux disease in adults: a pharmacist's perspective. Integr Pharm Res Pract.

[20] Multidisciplinary Branch of Gastroesophageal Reflux of China International Exchange and Promotion Association for Medical and Healthcare (2020). Multidisciplinary branch of gastroesophageal reflux of china international exchange and promotion association for medical and healthcare. Chin J Gastroesophagol Reflux Dis.

[21] Ong AM, Chua LT, Khor CJ, Asokkumar R, Son V, Wang YT (2018). Diaphragmatic breathing reduces belching and proton pump inhibitor refractory gastroesophageal reflux symptoms. Clin Gastroenterol Hepatol Off Clin Pract J Am Gastroenterol Assoc.

[22] Hansen JM, Wildner-Christensen M, Schaffalitzky de Muckadell OB (2009). Gastroesophageal reflux symptoms in a Danish population: a prospective follow-up analysis of symptoms, quality of life, and health-care use. Am J Gastroenterol.

[23] Müller-Stich BP, Linke GR, Senft J, Achtstätter V, Müller PC, Diener MK (2015). Laparoscopic Mesh-augmented hiatoplasty with cardiophrenicopexy versus laparoscopic nissen fundoplication for the treatment of gastroesophageal reflux disease: a double-center randomized controlled trial. Ann Surg.

[24] Revicki DA, Wood M, Wiklund I, Crawley J (1998). Reliability and validity of the gastrointestinal symptom rating scale in patients with gastroesophageal reflux disease. Qual Life Res Int J Qual Life Asp Treat Care Rehabil.

[25] Oh B, Kim BS, Kim JW, Kim JS, Koh SJ, Kim BG (2016). The Effect of probiotics on gut microbiota during the helicobacter pylori eradication: randomized controlled trial. Helicobacter.

[26] Zhong B (2009). How to calculate sample size in randomized controlled trial?. J Thorac Dis.

[27] Zhang J, Guo Z, Sun Z, Chen W, Zhang H (2011). The effect of probiotics on intestinal flora: taking the study of *Lactobacillus casei* Zhang for an example. J Chin Inst Food Sci Technol.

[28] Zhu H, Cao C, Wu Z, Zhang H, Sun Z, Wang M (2021). The probiotic *L. casei* Zhang slows the progression of acute and chronic kidney disease. Cell Metab.

[29] Ma C, Peng Q, Jiang S, Chen K, Fang Y, Zhang J (2018). Probiotic *Bifidobacterium lactis* V9 regulates the intestinal microbiome in patients with polycystic ovary syndrome. Chin Sci Bull.

