# Supplementary material for: Protocol of a randomized, double-blind, placebo-controlled study of the effect of probiotics on the gut microbiome of patients with gastro-oesophageal reflux disease treated with rabeprazole
Source: BMC Gastroenterol. 2022 May 20;22:255. doi: 10.1186/s12876-022-02320-y (PMC9123715; doi:10.1186/s12876-022-02320-y)
Supplement: Supplementary file 1 — Additional file 1: Appendix 1. Trial registration dataset. [file 12876_2022_2320_MOESM1_ESM.docx]

**Appendix 1 Trial registration-dataset**

| **Data category** | ChiCTR2000038409 |
| --- | --- |
| Primary registry and trial identifying number | http://www.chictr.org.cn/showproj.aspx?proj=56358 |
| Date of registration in follow-up registry | 5 November 2021 |
| Source(s) of monetary or material support | The trial was supported by the National Natural Science Foundation of China (31720103911) and Jiangzhong Pharmaceutical Company Limited to Heping Zhang. |
| Primary sponsor | Jiangzhong Pharmaceutical Co., Ltd. |
| Contact for public queries | SE, MD, MPH [email address] |
| Contact for scientific queries | SE, MD, MPH Bernhard Nocht Institute for Tropical Medicine, Hamburg, Germany |
| Public title | Effect of probiotics on gut microbiota in patients with gastroesophageal reflux treated with rabeprazole |
| Scientific title | *Effect of probiotics on gut microbiota in patients with gastroesophageal reflux treated with rabeprazole* |
| Countries of recruitment | China |
| Health condition(s) or problem(s) studied | Probiotics; Proton pump inhibitors; Gut microbiome |
| Intervention(s) | ***Probiotics group:*** *Initial Treatment Period (Weeks 1-8): Multiple probiotics+ rabeprazole. Maintenance Treatment Period (Weeks 9-12): Multiple probiotics.*  *Multiple probiotics containing strains such as Lactobacillus casei Zhang, Bifidobacterium lactis V9, and Lactobacillus plantarum P9, 2 g/bar, each containing active probiotics (≥ 50 billion CFU), were obtained from Jiangzhong Pharmaceutical Company Limited (China). Rabeprazole dosage: 10 mg/tablet, twice a day, before meals.*  ***Placebo group****: Initial Treatment Period (Weeks 1-8): Probiotic placebo + rabeprazole. Maintenance Treatment Period (Weeks 9-12): Probiotic placebo.*  *The Probiotic placebo ingredients are maltodextrin, orange powder, and maltitol, and there are no active ingredients. The appearance, packaging, storage method, and dosing were the same as those of the* *Multiple probiotics from Jiangzhong Pharmaceutical Company Limited (China).* |
| Key inclusion and exclusion criteria | **Inclusion criteria:** (1) One of the following criteria must be met:  1) In the past 3 months, gastroscopy performed at domestic tertiary hospitals showed oesophagitis (LA-A, LA-B or LA-C);  2) In the past 3 months, gastroscopy performed at domestic tertiary hospitals did not reveal oesophagitis, but symptoms such as heartburn, acid reflux, and poststernal burning pain are present; in addition, the RDQ score is ≥ 12;  (2) The subject is aged 18-65 years (inclusive) and is male or female;  (3) The subject has signed an informed consent form. |
|  | **Exclusion criteria:** (1) Used GERD-related drugs, such as acid inhibitors, antacids, prokinetics, gastric mucosal protectors, and herbs (see Appendix 3), or probiotics and probiotic-related preparations in the last 2 weeks;  (2) Any of the following conditions:  a. Liver insufficiency, defined as alanine aminotransferase (ALT) or aspartate aminotransferase (AST) > 2 × upper limit of normal (ULN);  b. Renal insufficiency, defined as serum creatinine (Scr) > 1 x ULN;  c. Heart failure or electrocardiogram (ECG) abnormalities;  (3) Peptic ulcer and bleeding, oesophageal gastric varices, or upper gastrointestinal malignancies confirmed by endoscopy at tertiary hospitals in China in the last 3 months;  (4) Myocardial infarction, stroke, or malignant tumour;  (5) History of gastro-oesophageal or duodenal surgery;  (6) Plans to become pregnant or father a child in the near future, or pregnancy or breastfeeding in  women.  (7) Inability to cooperate, such as an inability to understand the informed consent form or unwillingness to provide personal information;  (8) Allergies to the study drug (rabeprazole) or probiotics;  (9) Oesophagitis caused by gastric retention and pyloric obstruction. |
| Study type | Interventional |
|  | Allocation: randomized intervention model. Parallel assignment masking: double blind (subject, caregiver, investigator, outcomes assessor) |
|  | Primary purpose: prevention |
| **Date of first enrolment** | **September 2020** |
| Target sample size | 120 |
| Recruitment status | Recruiting |
| Primary outcome(s) | The primary measure is the change in gut microbiome. |
| Key secondary outcomes | The secondary measures are the Reflux Disease Questionnaire score, Gastrointestinal Symptom Rating Scale score, faecal metabolome, body mass index and Los Angeles grade of oesophagitis. |
