# Supplementary material for: Protocol of a randomized, double-blind, placebo-controlled study of the effect of probiotics on the gut microbiome of patients with gastro-oesophageal reflux disease treated with rabeprazole
Source: BMC Gastroenterol. 2022 May 20;22:255. doi: 10.1186/s12876-022-02320-y (PMC9123715; doi:10.1186/s12876-022-02320-y)
Supplement: Supplementary file 2 — Additional file 2: Appendix 2. Copy of Ethical Approval Document with translated English version. [file 12876_2022_2320_MOESM2_ESM.docx]

**Appendix 2 Copy of Ethical Approval Document with English translation**

**IRB opinion letter of the First Affiliated Hospital of Nanchang University**

Review opinion number: IIT [2020] Clinical Ethics Review No. 003-2

| Project name | A randomized, double-blind, placebo-controlled study of the effect of probiotics on the gut microbiome of patients with gastro-oesophageal reflux disease treated with rabeprazole | | |
| --- | --- | --- | --- |
| Sponsor | Jiangzhong Pharmaceutical Co., Ltd./The First Affiliated Hospital of Nanchang University | | |
| Category of research | investigator-initiated clinical trial | | |
| Specialty of Research | Gastroenterology | Principal Investigator | Nonghua Lu |
| Review time | September 16, 2021 | Review site | \ |
| Review form | Quick review | Review category | Amendment review |
| Review documents | | | |
| clinical trial protocol (Version no. 3.0 Version Date: 9 September 2021); Informed consent (Version no. 3.0 Version Date: 9 September 2021); Case Report Form (Version no. 3.0 Version Date: 9 September 2021) | | | |
| Review decisions | Approval, tracking review rate unchanged | | |
| Attention | 1. Any changes to the research data during the study should be submitted to the ethics committee for review and approval before implementation. 2. According to the suggestion of the ethics committee on the frequency of follow-up review, no matter whether the trial is started or not, please submit an application for follow-up review one month before the expiration of the follow-up review. After the research, please submit a final report as needed. 3. The approval document is valid for one year, if it is not implemented within the time limit, it will be automatically annulled. 4. If the project involves sending biological samples of human bodies to overseas laboratories for testing, the approval documents will be submitted to the Medical Ethics Committee for record after obtaining the approval of the “China Human Genetic Resources Management Office” before the relevant work can be carried out. | | |
| Chairman's signature:  Date: 17 September 2021  Medical Ethics Committee of the First Affiliated Hospital of Nanchang University ([seal](file:///C:/Users/13424/AppData/Local/youdao/dict/Application/8.9.6.0/resultui/html/index.html#/javascript:;)): | | | |
| Statement: The ethics committee is composed and working in strict accordance with GCP ICH-GCP and related regulations  Address: No. 17 Yongwaizheng Street, Nanchang City Contact: Sun Wenxiong, Tan Haoqu Tel & Fax: 0791-88692201 | | | |
