# Supplementary material for: Protocol of a randomized, double-blind, placebo-controlled study of the effect of probiotics on the gut microbiome of patients with gastro-oesophageal reflux disease treated with rabeprazole
Source: BMC Gastroenterol. 2022 May 20;22:255. doi: 10.1186/s12876-022-02320-y (PMC9123715; doi:10.1186/s12876-022-02320-y)
Supplement: Supplementary file 3 — Additional file 3: Appendix 3. Patient informed consent form. [file 12876_2022_2320_MOESM3_ESM.docx]

**Appendix 3 Patient informed consent form**

A randomized, double-blind, placebo-controlled study of the effect of probiotics on the gut microbiome of patients with gastro-oesophageal reflux disease treated with rabeprazole

Dear Patients,

You will be invited to participate in a clinical trial. This study is jointly sponsored by the First Affiliated Hospital of Nanchang University, Inner Mongolia Agricultural University, and Jiangzhong Pharmaceutical Co. Ltd. The project is called “A randomized, double-blind, placebo-controlled study of the effect of probiotics on the gut microbiome of patients with gastro-oesophageal reflux disease treated with rabeprazole”. The study details are described below:

**1. Study background and purpose**

Gastroesophageal reflux disease (GERD) is the result of reflux of gastroduodenal contents into the oesophagus, causing acid reflux, heartburn and the extra-oesophageal manifestations of asthma, chronic cough, idiopathic pulmonary fibrosis, hoarseness, laryngitis and odontosis. GERD is a common disease, and the prevalence rate in China is 5.77-7.28%; the prevalence rate of symptomatic GERD in China is 3.1%. Typical symptoms of GERD are heartburn and reflux, which can be accompanied by noncardiac chest pain, abdominal pain, upper abdominal pain, upper abdominal burning, belching and other atypical symptoms, which have a serious impact on patients' quality of life. Currently, acid suppressors (such as rabeprazole) are generally used for 8-12 weeks; however, they are associated with more adverse reactions when used for a long time, including adverse effects on the intestinal flora. Probiotics, a class of beneficial bacteria that can colonize the intestinal tract, can improve intestinal flora disorders and can improve reflux symptoms.

In this study, patients with gastroesophageal reflux symptoms who required long-term acid inhibitor treatment were selected and treated with rabeprazole plus probiotics to observe and explore the effect of probiotics on the intestinal microflora balance of GERD patients treated with rabeprazole. The effect of probiotics combined with rabeprazole on symptom improvement in patients with gastroesophageal reflux was also explored.

**2. Profiles of the probiotics**

The probiotics used in this study are Lihuo brand probiotics provided by Jiangzhong Pharmaceutical Co., Ltd. The strains include Lactobacillus casei Zhang, Bifidobacterium lactis V9, and Lactobacillus plantarum P9. Among them, Lactobacillus casei Zhang is a probiotic with excellent performance that is isolated and screened from traditional fermented kefir milk in Inner Mongolia; Bifidobacterium V9 is a probiotic isolated from healthy human intestines; and Lactobacillus plantarum P9 is isolated, naturally fermented sour porridge from Bayannaoer City. Early clinical studies showed that Lactobacillus casei Zhang, Bifidobacterium V9, and Lactobacillus plantarum P9 can regulate the intestinal flora, increase beneficial bacteria, and reduce harmful bacteria.

**3. The following conditions are required to participate in this study:**

The doctor or research team member in charge of this study will discuss the requirements to participate in this study with you. You need to provide a complete medical history to the study doctor or study team member. If you meet the following criteria, you will likely enter the screening period of this study based on the judgement of the study doctor, and if you meet all the criteria for participating in this study, you will enter the treatment period of this study. You can participate in this study only if the following conditions are met:

**Inclusion criteria**

(1) One of the following criteria must be met:

1) In the past 3 months, gastroscopy performed at domestic tertiary hospitals has shown oesophagitis (LA-A, LA-B or LA-C);

2) In the past 3 months, gastroscopy performed at domestic tertiary hospitals has not revealed oesophagitis, but you have symptoms such as heartburn, acid reflux, and poststernal burning pain; in addition, the RDQ score is ≥ 12;

(2) The subject’s age is 18-65 years (inclusive), and he or she is male or female;

(3) The subject signed an informed consent form.

Exclusion criteria

(1) Use of GERD-related drugs such as acid inhibitors, antacids, prokinetics, gastric mucosal protectors, and herbs (see Appendix 3) or probiotics and probiotic-related preparations in the last 2 weeks;

(2) Any of the following conditions:

1) Liver insufficiency, defined as alanine aminotransferase (ALT) or aspartate aminotransferase (AST) > 2 × upper limit of normal (ULN);

2) Renal insufficiency, defined as serum creatinine (Scr) > 1 x ULN;

3) Heart failure or electrocardiogram (ECG) abnormalities;

(3) Peptic ulcer and bleeding, oesophageal gastric varices, or upper gastrointestinal malignancies confirmed by endoscopy at tertiary hospitals in China in the last 3 months;

(4) Myocardial infarction, stroke, or malignant tumour;

(5) History of gastro-oesophageal or duodenal surgery;

(6) Plans to become pregnant or father a child in the near future, or pregnancy or breastfeeding in women.

(7) Inability to cooperate, such as an inability to understand the informed consent form or unwillingness to provide personal information;

(8) Allergies to the study drug (rabeprazole) or probiotics;

(9) Oesophagitis caused by gastric retention and pyloric obstruction.

**4. Research stage and steps**

If you consent to participate in this study, please sign this informed consent form. One blood collection was planned, and stool was collected four times throughout the study period.

(1) Before treatment, the doctor will ask and record your medical history and conduct abdominal B-ultrasound, electrocardiography, routine blood, blood biochemistry and routine stool screening examinations. If you meet the conditions for enrolment, you will enter the study and receive drugs and probiotics/probiotic placebo later.

(2) The treatment period includes the initial treatment period (weeks 1-8) and maintenance treatment period (weeks 9-12). There are 3 follow-up visits, for which stool samples should be collected and two scoring questionnaire forms should be completed.

The entire study process was divided into screening and treatment periods, including the initial treatment period (weeks 1-8) and the maintenance treatment period (weeks 9-12). During each treatment cycle, you need to cooperate with the doctor's examination and treatment. Your doctor will ask you regularly according to the provisions of the research plan to evaluate the efficacy and safety of the test drug treatment.

**5. Treatment group**

The study will be divided into trial and control groups, with patients randomly assigned to one of the following two groups, and randomly assigned to the control or trial group. There are risks, discomfort, and inconveniences in any scientific study. If you have any adverse reactions, the clinician will provide timely treatment.

- Initial Treatment Period (Weeks 1-8)

Control group: rabeprazole + probiotic placebo

Test group: rabeprazole + probiotics

- Maintenance Treatment Period (Weeks 9-12)

Control group: Probiotic placebo

Test group: probiotics

Dosage: Rabeprazole (Eisai Pharmaceutical Co., LTD.), 10 mg/time, twice a day, before meals. Probiotic placebo or Lihuo brand probiotics: 2 capsules/time, once per day (100 billion CFU/day), taken orally directly or with warm water (below 40°C), taken after meals, and taken 2 h after antibiotic ingestion if applicable.

**6. Possible benefits from participating in the study**

Your gastroesophageal reflux condition is likely to be improved by participating in this trial. The expected improvement may include the reduction or elimination of gastroesophageal reflux symptoms and reduction of the unhealthy situation of intestinal flora caused by rabeprazole, as well as of gastrointestinal symptoms (e.g., abdominal pain and discomfort, abdominal distension, nausea, vomiting, belching, diarrhoea, and constipation); however, we cannot guarantee that you will benefit from the study.

Participants will receive 2 months of rabeprazole for free and free examinations related to the protocol. In addition, at the beginning of the study, we will provide each person with a weight scale and tape measure, and at the end of the study, we will provide a transportation subsidy of 100 yuan per person.

**7. Participation in the study/midway withdrawal/study termination**

Participation in the study depends entirely on your willingness. You may refuse to participate in this study or withdraw from this study at any time during the study, which will not affect your relationship with your doctor and cause no loss of your medical or other benefits.

**8. Alternative treatment protocol**

In addition to participating in this study, you can choose to receive routine treatment provided by your doctor, such as an acid suppressor. You can discuss other treatment options with your doctor.

**9. Confidentiality in the trial**

If you agree to participate in this study, your medical records will be reviewed by the executors and supervisors of the study. All information collected from you during the study is kept strictly confidential, and only your contact information is listed in a form containing identifiable information. We will keep the form in a secure database used to contact you by telephone in the future. However, during the data analysis process, all your data will be removed, and no personal information will be disclosed in later publications or other published articles.

**10 Risk and discomfort in the trial**

During the period of rabeprazole administration, you may have allergic reactions (including skin rash, itchy skin, hives), digestive system abnormalities (constipation, diarrhoea, abdominal distension, nausea, lower abdominal pain, pain in the mouth), headache, and dizziness. Ingestion of probiotics may cause a gurgling sound in the stomach and the production of slightly more gas. Existing studies have shown that people who are intolerant to probiotics can experience diarrhoea. This reaction does not usually last long and resolves for most people within a few days to 2 weeks.

Any scientific research has risks, discomforts, and inconveniences, which you should consider thoroughly before agreeing to participate in any clinical research.

**11. Trial cost**

During the clinical study period, the sponsor will provide free study drugs, Lihuo brand probiotics and a probiotic placebo until the end of the trial and cover the examination costs specified by the protocol during the study period. During the study period, if there are serious adverse reactions related to the study drug, Jiangzhong Pharmaceutical Co., Ltd., will provide reimbursement of the corresponding treatment expenses and corresponding economic compensation for the study-related damage in accordance with the relevant laws and regulations of China.

**Signature page**

**If you or your family/guardian agree to participate in this study, please read and sign.**

I have been informed of the purpose and method of this trial and the possible risks, discomforts, and related benefits.

I am sure I have spent enough time to read and understand the above, the investigator has explained the medical terms used in the above, and the investigator has provided satisfactory answers to my questions related to this study. I understand that I can voluntarily withdraw from this study at any time without affecting future doctor-patient relationships and treatment. I know that if I have any questions during the trial, I should contact the researcher in a timely manner.

I volunteered for this trial and served as a subject in this trial.

Signature of subject or designated agent

Signature date year month day

I have truthfully informed the subject (or designated agent) of the purpose, content, benefits, and possible adverse reactions associated with this research. I have asked if they have any questions about this research and have tried my best to explain it.

Investigator signature

Signature date year month day
