# Supplementary material for: Protocol of a randomized, double-blind, placebo-controlled study of the effect of probiotics on the gut microbiome of patients with gastro-oesophageal reflux disease treated with rabeprazole
Source: BMC Gastroenterol. 2022 May 20;22:255. doi: 10.1186/s12876-022-02320-y (PMC9123715; doi:10.1186/s12876-022-02320-y)
Supplement: Supplementary file 4 — Additional file 4: Appendix 4. Common Drugs Taken for Gastroesophageal Reflux SymptomsAppendix. [file 12876_2022_2320_MOESM4_ESM.docx]

**Appendix 4 Common Drugs Taken for Gastro-oesophageal Reflux Symptoms**

| **Type of Drug** | **Drug Name** |
| --- | --- |
| PPIs | Omeprazole, esomeprazole, lansoprazole, pantoprazole, rabeprazole, ilaprazole, esomeprazole, tenatoprazole |
| Histamine H2 antagonists | Cimetidine, ranitidine, famotidine, roxatidine, nizatidine |
| Antacids | Aluminium magnesium carbonate, aluminium phosphate gel, sodium bicarbonate, calcium carbonate, aluminium hydroxide, magnesium hydroxide, magnesium trisilicate |
| Gastric mucosal protectors | Sucralfate, rebamipide, teprenone, gemfaxate, misoprostol, hydrotalcite, aluminium magnesium plus, bismuth potassium citrate, colloidal pectin bismuth, marzulene-S, Kangfuxin solution, irsogladine maleate, L-glutamine sodium gualenate, alginic acid (sodium alginate) |
| Prokinetics | Metoclopramide, domperidone, itopride, mosapride, trimebutine maleate |
| Herbs/herbal formulations | Zhizhu Kuanzhong capsules, Weisu granules, Da li tong granules, Jiawei Zuojin pills, Nunling Weitong granules, Jinghua Weikang capsules, Qi Stagnation Weitong granules, Shugan Jieyu capsules, Heart Stomach Pain capsules, Xiangsha Pingwei granules, Chaihu Shugan powder and Zuojin pills, Xiao Chaihu decoction and Wendan decoction, Xuanfu Daizhe decoction and Liujunzi decoction, Banxia Houpu decoction, Banxia Xiexin decoction |
