# Supplementary material for: Protocol of a randomized, double-blind, placebo-controlled study of the effect of probiotics on the gut microbiome of patients with gastro-oesophageal reflux disease treated with rabeprazole
Source: BMC Gastroenterol. 2022 May 20;22:255. doi: 10.1186/s12876-022-02320-y (PMC9123715; doi:10.1186/s12876-022-02320-y)
Supplement: Supplementary file 5 — Additional file 5: Appendix 5. Reflux Disease Questionnaire. [file 12876_2022_2320_MOESM5_ESM.docx]

**Appendix 5 Reflux Disease Questionnaire (RDQ)**

**Please read each statement and select the response that best describes how much the statement applied to you over the past week. Please try to answer every question. There are no right or wrong answers.**

| **Symptom** | **By weekly frequency** | **By symptom severity** |
| --- | --- | --- |
| **Heartburn** |  0: None;   1: < 1 day a week;   2: 1 day a week;   3: 2-3 days a week;   4: 4-5 days a week;  5: 6-7 days a week. |  0: None;   1: The symptom is barely noticeable and was identified by a doctor;   2: Between 1 and 3 in severity;   3: Apparent symptom that affects daily activities and sometimes requires medications;   4: Between 3 and 5 in severity;  5: Significant symptom that affects daily activities and requires long-term medications; |
| **Regurgitation** |  0: None;   1: < 1 day a week;   2: 1 day a week;   3: 2-3 days a week;   4: 4-5 days a week;  5: 6-7 days a week; |  0: None;   1: The symptom is barely noticeable and was identified by a doctor;   2: Between 1 and 3 in severity;  3: Apparent symptom that affects daily activities and sometimes requires medications;   4: Between 3 and 5 in severity;  5: Significant symptom that affects daily activities and requires long-term medications. |
| **Acid reflux** |  0: None;   1: < 1 day a week;   2: 1 day a week;   3: 2-3 days a week;   4: 4-5 days a week;  5: 6-7 days a week; |  0: None;   1: The symptom is barely noticeable and was identified by a doctor;   2: Between 1 and 3 in severity;   3: Apparent symptom that affects daily activities and sometimes requires medications;   4: Between 3 and 5 in severity;  5: Significant symptom that affects daily activities and requires long-term medications. |
| **Noncardiac chest pain** |  0: None;   1: < 1 day a week;   2: 1 day a week;   3: 2-3 days a week;   4: 4-5 days a week;  5: 6-7 days a week; |  0: None;   1: The symptom is barely noticeable and was identified by a doctor;   2: Between 1 and 3 in severity;  3: Apparent symptom that affects daily activities and sometimes requires medications;   4: Between 3 and 5 in severity;  5: Significant symptom that affects daily activities and requires long-term medications. |
