# Supplementary material for: Protocol of a randomized, double-blind, placebo-controlled study of the effect of probiotics on the gut microbiome of patients with gastro-oesophageal reflux disease treated with rabeprazole
Source: BMC Gastroenterol. 2022 May 20;22:255. doi: 10.1186/s12876-022-02320-y (PMC9123715; doi:10.1186/s12876-022-02320-y)
Supplement: Supplementary file 6 — Additional file 6: Appendix 6. Gastrointestinal Symptom Rating Scale. [file 12876_2022_2320_MOESM6_ESM.docx]

**Appendix 6 Gastrointestinal Symptom Rating Scale (GSRS)**

**Please read each statement and select the response that best describes how much the statement applied to you over the past week. Please try to answer every question according to the degree of symptoms, frequency, duration, mitigating factors and impact on social activities. There are no right or wrong answers.**

| **Symptom** | | A**ccording to the degree of symptoms, frequency, duration, mitigating factors and impact on social activities** |
| --- | --- | --- |
| **Abdominal pain syndrome** | **Abdominal pain** | 0 No or transient pain  1 Occasional aches and pains interfering with  some social activities  2 Prolonged and troublesome aches and pains  causing requests for relief and interfering with  many social activities  3 Severe or crippling pain with an impact on all  social activities |
|  | **Sucking sensations in the epigastrium** | 0 No or a transient sucking sensation  1 Occasional discomfort of short duration; no  requests for food or antacids between meals  2 Frequent episodes of prolonged discomfort;  requests for food and antacids between meals  3 Continuous discomfort; frequent requests for  food or antacids between meals |
|  | **Nausea and vomiting** | 0 No nausea  1 Occasional episodes of short duration  2 Frequent and prolonged nausea; no vomiting  3 Continuous nausea; frequent vomiting |
| **Dyspeptic syndrome** | **Acid regurgitation** | 0 No or transient regurgitation  I Occasional troublesome regurgitation  2 Regurgitation once or twice a day; requests for  relief  3 Regurgitation several times a day; only transient  and insignificant relief with antacids |
|  | **Heartburn** | 0 No or transient heartburn  1 Occasional discomfort of short duration  2 Frequent episodes of prolonged discomfort;  requests for relief  3 Continuous discomfort with only transient relief  with antacids |
| **Indigestion syndrome** | **Abdominal distension** | 0 No or transient distension  1 Occasional discomfort of short duration  2 Frequent and prolonged episodes that can be  resolved by adjusting the clothing  3 Continuous discomfort seriously interfering  with social performance |
|  | **Eructation** | 0 No or transient eructation  1 Occasional troublesome eructation  2 Frequent episodes interfering with some social  activities  3 Frequent episodes seriously interfering with  social performance |
|  | **Increased flatus** | 0 No increased flatus  1 Occasional discomfort of short duration  2 Frequent and prolonged episodes interfering  with some social activities  3 Frequent episodes seriously interfering with  social performance |
|  | **Borborygmus** | 0 No or transient borborygmus  1 Occasional troublesome borborygmus of short  duration  2 Frequent and prolonged episodes that can be  resolved by movement without impairing social  performance  3 Continuous borborygmus severely interfering  with social performance |
| **Diarrhoea** | **Increased passage of stools** | 0 Once a day  I Three times a day  2 Five times a day  3 Seven times a day or more frequently |
|  | **Loose stools** | 0 Normal consistency  1 Somewhat loose  2 Runny  3 Watery |
|  | **Urgent need for defecation** | 0 Normal control  1 Occasional feelings of an urgent need for defecation  2 Frequent feelings of an urgent need for defecation, with sudden needs to use a toilet that interfere with  social performance  3 Inability to control defecation |
| **Constipation** | **Decreased passage of stools** | 0 Once a day  1 Every third day  2 Every fifth day  3 Every seventh day or less frequently |
|  | **Hard Stools** | 0 Normal consistency  1 Somewhat hard  2 Hard  3 Hard and fragmented, sometimes in combination  with diarrhoea |
|  | **Feeling of incomplete evacuation** | 0 Feeling of complete evacuation without straining  1 Defecation that is somewhat difficult; occasional feelings  of incomplete evacuation  2 Defecation definitely difficult; frequent feelings of  incomplete evacuation  3 Defecation extremely difficult; regular feelings  of incomplete evacuation |
