# Supplementary material for: Protocol of a randomized, double-blind, placebo-controlled study of the effect of probiotics on the gut microbiome of patients with gastro-oesophageal reflux disease treated with rabeprazole
Source: BMC Gastroenterol. 2022 May 20;22:255. doi: 10.1186/s12876-022-02320-y (PMC9123715; doi:10.1186/s12876-022-02320-y)
Supplement: Supplementary file 7 — Additional file 7: Appendix 7. National Natural Science Foundation of China with translated English version [file 12876_2022_2320_MOESM7_ESM.docx]

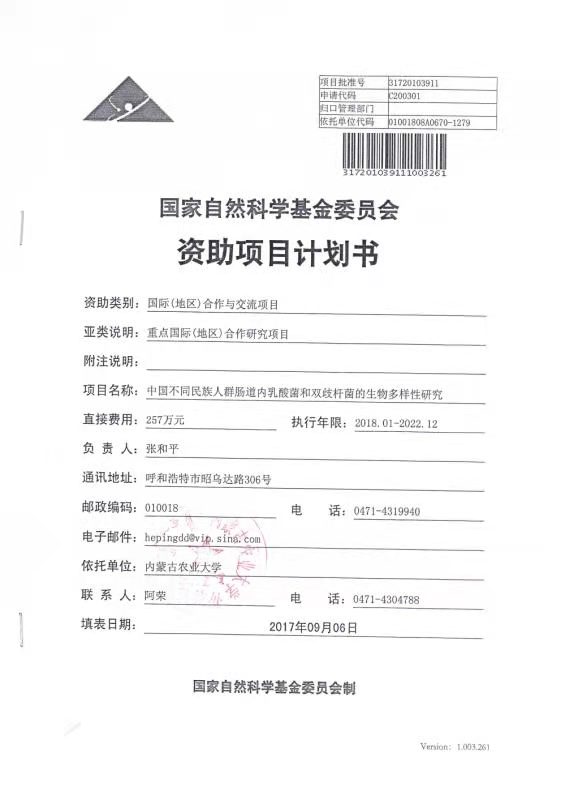


**Appendix 7 Copy of National Natural Science Foundation of China with English translation**

Project approval number: 31720103911

Application code: C200301

Central Management Department:

Relying unit code: 01001808A0670-1279

National Natural Science Foundation of China

Funding project plan

Funding category: International (regional) cooperation and exchange projects

Subcategory description: Key international (regional) cooperative research projects

Note description:

Project name: Study on Biodiversity of *Lactobacillus* and *Bifidobacterium* in the Intestines of Different Ethnic Groups in China

Direct cost: ¥2.57 million Execution period: January 2018 to December 2022

Person in charge: Heping Zhang

Mailing address: No. 306, Zhaowuda Road, Hohhot

Postal Code: 010018 Phone: 0471-439940

Email: hepingdd@vip.sina.com

Supporting unit: Inner Mongolia Agricultural University

Contact: A Rong Phone: 0471-4304788

Date of completion: September 06, 2017

National Natural Science Foundation of China


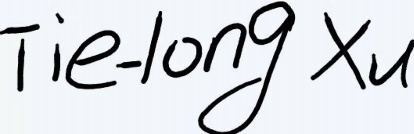


Signature:
